# The Role of Adhesion Molecules as Biomarkers for the Aggressive Prostate Cancer Phenotype

**DOI:** 10.1371/journal.pone.0081666

**Published:** 2013-12-16

**Authors:** Claire Morgan, Spencer A. Jenkins, Howard G. Kynaston, Shareen H. Doak

**Affiliations:** 1 Cancer Biomarkers Group, Institute of Life Science, College of Medicine, Swansea University, Swansea, United Kingdom; 2 Department of Urology, University Hospital of Wales, Heath Park, Cardiff, United Kingdom; Aix-Marseille University, France

## Abstract

**Background:**

Currently available methods for diagnosis and staging of prostate cancer lack the sensitivity to distinguish between patients with indolent prostate cancer and those requiring radical treatment. Alterations in key adherens (AJ) and tight junction (TJ) components have been hailed as potential biomarkers for prostate cancer progression but the majority of research has been carried out on individual molecules.

**Objective:**

To elucidate a panel of biomarkers that may help distinguish dormant prostate cancer from aggressive metastatic disease.

**Methods:**

We analysed the expression of 7 well known AJ and TJ components in cell lines derived from normal prostate epithelial tissue (PNT2), non-invasive (CAHPV-10) and invasive prostate cancer (LNCaP, DU145, PC-3) using gene expression, western blotting and immunofluorescence techniques.

**Results:**

Claudin 7, α –catenin and β-catenin protein expression were not significantly different between CAHPV-10 cells and PNT2 cells. However, in PC-3 cells, protein levels for claudin 7, α –catenin were significantly down regulated (−1.5 fold, p = <.001) or undetectable respectively. Immunofluoresence showed β-catenin localisation in PC-3 cells to be cytoplasmic as opposed to membraneous.

**Conclusion:**

These results suggest aberrant Claudin 7, α – and β-catenin expression and/or localisation patterns may be putative markers for distinguishing localised prostate cancer from aggressive metastatic disease when used collectively.

## Introduction

Prostate cancer (CaP) is the most common cancer in men in the UK. Every year nearly 35,000 cases of CaP are diagnosed and it accounts for approximately 12% of all male deaths from cancer in the UK [1]. Early diagnosis of organ-confined CaP is essential since radical prostatectomy or radiotherapy offers the only chance of complete cure and treatment of advanced disease is palliative and in-effective (in the long term). In addition, early organ-confined CaP is not always life threatening and can follow and indolent course which does not require treatment. It is generally accepted that currently available methods used for diagnosis and staging of CaP (prostate specific antigen levels, gleason score and clinical and pathological grade) lack the sensitivity to distinguish between patients with indolent, organ-confined CaP, those requiring radical treatment and those at risk of relapse after radical treatment. Clearly there is a need to identify molecular markers of CaP progression, invasion and metastasis to predict diagnosis and guide therapy.

For a cancer to metastasise, cells must first break away from the primary tumour. In epithelial tissue, cells are connected to one another by membrane structures called tight junctions (TJ), adherens junctions (AJ) and desmosomes [2]. Together they maintain the architecture of the epithelium. AJ proteins comprise the cadherin and catenin families. E-cadherin is primarily present in epithelia and represents the prototypic member of the cadherin family. The cytoplasmic domain of E-cadherin binds to cytosolic proteins called catenins (α, β and p120) [3]. β-catenin binds directly to E-cadherin while α-catenin binds indirectly via its interaction with β-catenin [4]. Together with desmosomes they are primarily responsible for adhesion between adjacent cells [5] and forming stable cell-cell contacts. The TJ separate the apical and basolateral regions of the plasma membrane and regulate the passage of ions, water and macromolecules across the epithelium [6]. TJs are made up of membrane-bound proteins (claudins, occludin, tricellulin) and their adapter and scaffolding proteins (junctional adhesion molecule, ZO-1, ZO-2, ZO-3 cingulin, MUPP1) [7].

Until recently, TJs have only been perceived as cellular seals [8]. Now, however, losses of TJ proteins are being recognised for their association with a variety of cancers. Deregulation of TJ proteins is associated with the loss of epithelial cell polarity and dedifferentiation which is a known event in early stage carcinogenesis [7]. In poorly differentiated breast [9], [10], thyroid [11], endometrial [12] and gastrointestinal [13] cancers the expression of tight junction proteins have been shown to be reduced, while in breast cancer patients' loss of functional TJ proteins has also been associated with a poorer prognosis [14], [15]. Since TJ proteins are defined as the point where the membranes of two cells join together they perform a vital function by holding cells together and are a crucial barrier that cancer cells must overcome in order to spread [16].

While evidence continues to grow regarding the expression of TJ and AJ components in cancer the majority of studies are aimed at investigating individual molecules. Cancers are heterogeneous by nature and thus, it is impossible to diagnose cancer or predict disease progression using a single biomarker.

We utilised five commercially available prostate cell lines derived from normal prostate epithelium, non-invasive prostate cancer and metastatic cancer to analyse the expression of 7 well known AJ and TJ components, identified as aberrantly expressed in prostate cancer tissue samples [17], [18], [19], [20], [21], in the first step of potentially elucidating a panel of biomarkers that may distinguish indolent cancer from aggressive metastatic disease when used collectively.

## Materials and Methods

### Cell culture

Prostate epithelial cells (PNT2) and prostate cancer cells derived from cancer metastatic to the lymph nodes (LNCaP) and bone (PC-3) were obtained from the European Collection of Cell Cultures. Prostate cancer cells derived from non-invasive prostate cancer (CAHPV-10) and cancer metastatic to the brain (DU145) were purchased from the American Type Culture Collection. PNT2, LNCaP, DU145 and PC-3 were routinely cultured in RPMI 1640, 2 mM L-Glutamine Glutamine and 10% (v/v) foetal calf serum (Invitrogen, Paisley UK). CAHPV-10 cells were cultured in Keratinocyte serum-free medium with 5 ng/ml human recombinant epidermal growth factor and 50 µg/ml bovine pituitary extract (Invitrogen, Paisley UK).

Cells were grown in a humidified atmosphere of 5% CO_2_ at 37°C. Cells were sub-cultured using 0.25% trypsin/EDTA (Sigma-Aldrich, Dorset, UK).

### Antibodies

Primary antibodies for mouse ZO-1 and rabbit Occludin, Claudin-1 and Claudin-7 were purchased from Invitrogen (Paisley, UK). Rabbit, α-catenin, β-catenin, E-cadherin and rabbit β-actin were purchased from New England Biolabs (Hertfordshire, UK). Horseradish peroxidise conjugated secondary anti-mouse/anti-rabbit antibodies were also purchased from New England Biolabs (Hertfordshire, UK).

For immunofluoresence, additional primary antibodies for α-catenin, β-catenin were obtained from Abcam (Cambridge, UK). Anti-mouse (Qdot655) and anti-rabbit (Qdot525) secondary antibodies were obtained from Invitrogen (Paisley UK).

### Gene expression analysis

Total cellular RNA was extracted using the RNeasy extraction kit (Qiagen, Crawley, UK) and residual DNA was removed by treating with DNA-free™ (Ambion, Cambridgeshire, UK) according to the manufacturers' instructions. cDNA was synthesised from 1 µg RNA using random primers and the High Capacity cDNA synthesis reverse transcription kit (Applied Biosystems, Warrington, UK). Real-time PCR reactions were performed in the iCycler iQ Thermal Cycler (Bio-Rad Laboratories, Hemel Hempstead, UK) using the SYBR Green detection methodology. Primer sets (Table 1.) were designed to be exon-exon spanning in order to minimise the possibility that any contaminating genomic DNA would be amplified. The primer sets used were also tested to ensure they all demonstrated equally amplification efficiencies. All reactions were performed in triplicate with 2 µl cDNA, 12.5 µl iQ SYBR Green Supermix (Bio-Rad Laboratories, Hemel Hempstead, UK) and 0.2 µM forward and reverse primers, in a final reaction volume of 25 µl. β-actin and HPRT were used as reference genes. PCR amplification conditions were 95°C for 3 min, followed by 40 cycles of 94°C for 30 s, 60°C for 30 s and 72°C for 30 s. Fold expression was normalised against the normal prostate epithelial PNT2 cells.

**Table 1:** Real-time PCR primer sequences.

Gene	Forward Primer (5′-3′)	Reverse Primer (5′-3′)
Occludin	GAGTACATGGCTGCTGCTGA	GCTCTTTAACTGCTTGCAATGAT
ZO-1	GGGAGGGTGAAGTGAAGACA	GATCTGAAGAGGCCATGGAA
Claudin 1	CGATGAGGTGCAGAAGATGA	CATTGACTGGGGTCATAGGG
Claudin 7	TGGCCATCAGATTGTCAAGA	AGGACAGGAACAGGAGAGCA
α-catenin	CTGGGAGGAGAGCTCATCA	TTTCACTGTTTGCACTACAGCATTC
β-catenin	TGTTCTCAGATTTCTGGTTGTT	CACTTTCTGAGATACCAGCC
E-cadherin	CTGTCGAAGCAGGATTGCAAA	GAAGAAACAGCAAGAGCAGCA
β-actin	GATGGCCACGGCTGCTTC	TGCCTCAGGGCAGCGGAA
HPRT	GACTGTAGATTTTATCAGACTGA	TGGATTATACTGCCTGACCAA

### Western blotting

Total protein was extracted using RIPA buffer (Sigma Aldrich, Dorset, UK). Thirty micrograms of protein was run on either 7.5% or 12% tris-glycine PAGE gels (Bio-Rad Laboratories, Hemel Hempstead, UK) depending on the size of the protein of interest. Proteins were transferred onto nitrocellulose membrane (Amersham Pharmacia Biotech, Amersham, UK). The membranes were blocked with 5% non fat dry milk in tween/TBS to inhibit non specific binding (all Sigma Aldrich, Dorset, UK) and incubated overnight at 4°C using primary antibodies to, Occludin (1∶83), ZO-1 (1∶250), Claudin-1 (1∶125), Claudin-7 (1∶125) α-catenin (1∶1000), β-catenin (1∶1000) and E-cadherin (1∶500). β-actin (1∶1000) was used as a control for protein loading. Membranes were washed free of primary antibody and incubated with horseradish peroxidase conjugated secondary anti-mouse/anti-rabbit antibodies (1∶1000) for 1 hr at room temperature. Proteins were visualized using the Immun-Star WesternC chemiluminescene detection kit (Bio-Rad Laboratories, Hemel Hempstead, UK) and relative expression was quantified using densitometry and the Quantity One software programme (Bio-Rad Laboratories, Hemel Hempstead, UK). Western blots were performed in duplicate.

### Immunofluorescence

Following mRNA and protein analysis, Claudin 7 α-catenin and β-catenin appeared to have similar expression levels to normal prostate cells which became altered in cells derived from an aggressive metastatic tumour. Therefore, immunofluoresence was only performed on these molecules to ascertain whether alterations in protein expression were followed by aberrant localisation. Cells were seeded at 1×10^5^ cells/ml onto sterile microscope slides contained in sterile quadriperm tissue culture plates (Invitrogen, Paisley UK). Cells were left overnight to adhere to the slides and grown to confluence for 5 days. Once cells were confluent media was removed, slides were washed twice with sterile phosphate buffered saline (PBS) (Invitrogen, Paisley UK) and fixed in 4% paraformaldehyde (PFA) (Sigma Aldrich, Dorset, UK) for 15 mins at room temperature. PFA was removed by washing the slides in PBS/100 mM glycine (Sigma Aldrich, Dorset, UK) 5 min three times. Cells were permeablised in 0.1% Triton X-100 in PBS for 10 minutes and washed in PBS three times. To quench the reactive groups following PFA fixation, slides were incubated in sodium borohydride (Fisher Scientific, Leicestershire, UK) at a concentration of 1 mg/ml for 10 minutes. This was performed three times followed by a 5 minute PBS wash before blocking the slides in 10% bovine serum albumin (BSA)/PBS for 30 minutes. Blocking solution was removed by performing 3×2 minute washes in PBS. Slides were incubated with anti-Claudin 7 (1.100 in 1%BSA/PBS), α-catenin and β-catenin (1∶50 1%BSA/PBS). Slides were incubated overnight at 4°C. Primary antibody was removed by 3×5 min washes, followed by incubation with secondary antibodies (1∶100 in 6%BSA/PBS) for 1 hr at room temperature. Secondary antibodies were removed by 3×5 min in PBS before slides were washed in water (2 min) 70%, 85% and 95% ethanol (2 min each). Slides were analysed using an AxioCam fluorescent microscope (Carl Zeiss Ltd, Hertfordshire, UK).

Negative controls (to rule out autofluorescence) were carried out by substituting the primary antibody for 1%BSA/PBS.

### Statistical analysis

Analysis carried out using the parametric ANOVA test. Dunnett's post hoc analysis was performed to compare cancer cells with the normal epithelial PNT2 control cells and Tukey post hoc analysis was performed for multiple group comparisons. A *p*-value<0.05 was considered significant.

NOTE: Only differences in gene expression and protein levels that satisfy both the p-value and a biological fold-change of <1.5> would be deemed a significant change.

## Results

### Gene Expression Analysis

**Figure 1:**
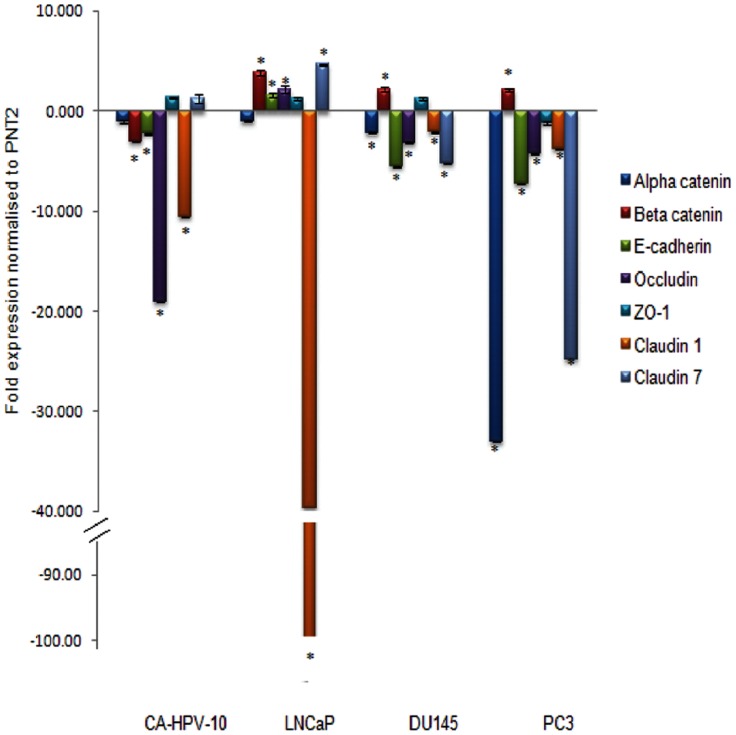
Differential mRNA expression of tight junction (Occludin, ZO-1, Claudin 1, Claudin 7) and adherence junctions (α- catenin, β-catenin and E-cadherin) in prostate cancer cell lines from localised (CAHPV-10) and different metastatic sites (LNCaP metastatic to lymph nodes, DU145 metastatic to the brain and PC-3 metastatic to bone). Expression levels have been normalised to the normal prostate cell line PNT2 whose expression level has been set to 1. * denotes significant difference (P<0.05) compared to PNT2.

Quantitative real-time PCR (Fig. 1) showed occludin mRNA levels were significantly different between all groups (*p*<0.001). A significant reduction in occludin mRNA levels was observed in CAHPV-10 (−19.08 fold, *p* = 0.001), DU145 (−3.2 fold, *p* = 0.003) and PC-3 (−4.3 fold, *p* = 0.003) prostate cancer cells compared to normal prostate epithelial cells (PNT2). There was no significant difference in occludin mRNA expression between the cancer cells regardless of invasive capacity. However, the mRNA levels were significantly up regulated (2.2 fold, *p* = 0.001) in the LNCaP cancer cells compared to prostate epithelial cells (PNT2).

With regards to ZO-1, there was no significant difference in transcriptional levels between normal prostate epithelial cells and any of the prostate cancer cells.

Claudin 1 gene expression was significantly down regulated in all the cancer cells compared to PNT2. In CAHPV-10 and LNCaP prostate cancer cells, Claudin 1 was down regulated −10 fold (*p* = <0.001) and −100-fold (*p* = <0.001) respectively. DU145 showed a −2.1-fold down regulation (*p* = <0.001) and PC-3 demonstrated a −3.8 fold (*p* = <0.001) reduced expression level.

Claudin 7 gene expression levels were similar between PNT2 and CAHPV-10 (expression value 1.1). In contrast, Claudin 7 transcription was significantly up regulated in LNCaP, (4.63 fold, *p* = <0.001), but down regulated in DU145, (−5.3 fold, *p* = 0.004) and PC-3, (−24.8 fold, *p* = 0.01) when compared to PNT2. There was also a significant down regulation in expression levels when Claudin 7 mRNA levels were compared between CAHPV-10 and (DU145, (*p* = 0.02) and PC-3 (*p* = <0.001).

Transcriptional levels for α- catenin did not differ significantly between PNT2, CAHPV-10, (−1.1 fold) or LNCaP,(−1 fold). A significant down regulation was observed by −2.2 fold in DU145 and −33 fold in PC-3 cells, (both p = <0.0001). When mRNA levels for CAHPV-10 were compared to DU145 and PC-3, a significant down-regulation in expression was detected (both DU145 and PC-3 *p* = <0.001).

β-catenin gene expression was significantly down-regulated in CAHPV-10 cells, (−3 fold, *p* = 0.015) compared to PNT2 while a significant up-regulation was observed in LNCaP, (3.8 fold, *p* = <0.001), DU145, (2.2 fold, *p* = 0.001) and PC-3, (2.1 fold, *p* = 0.001). There was also a significant up-regulation in expression levels when mRNA levels for CAHPV-10 were compared to DU145 (*p* = 0.006) and PC-3 (*p* = 0.001).

E-cadherin gene expression levels were significantly decreased in CAHPV-10 (−2.3 fold, *p* = 0.004), DU145 (−5.6 fold, *p* = <0.001) and PC-3 (−7.25 fold, *p* = <0.001) compared to PNT2. There was no significant difference in E-cadherin gene expression levels between the non-invasive and invasive prostate cancer cells. In contrast, the LNCaP cells once again demonstrated a significant up-regulation of E-cadherin as compared to PNT2 (1.6 fold, *p* = 0.037).

### Protein Expression Analysis

**Figure 2:**
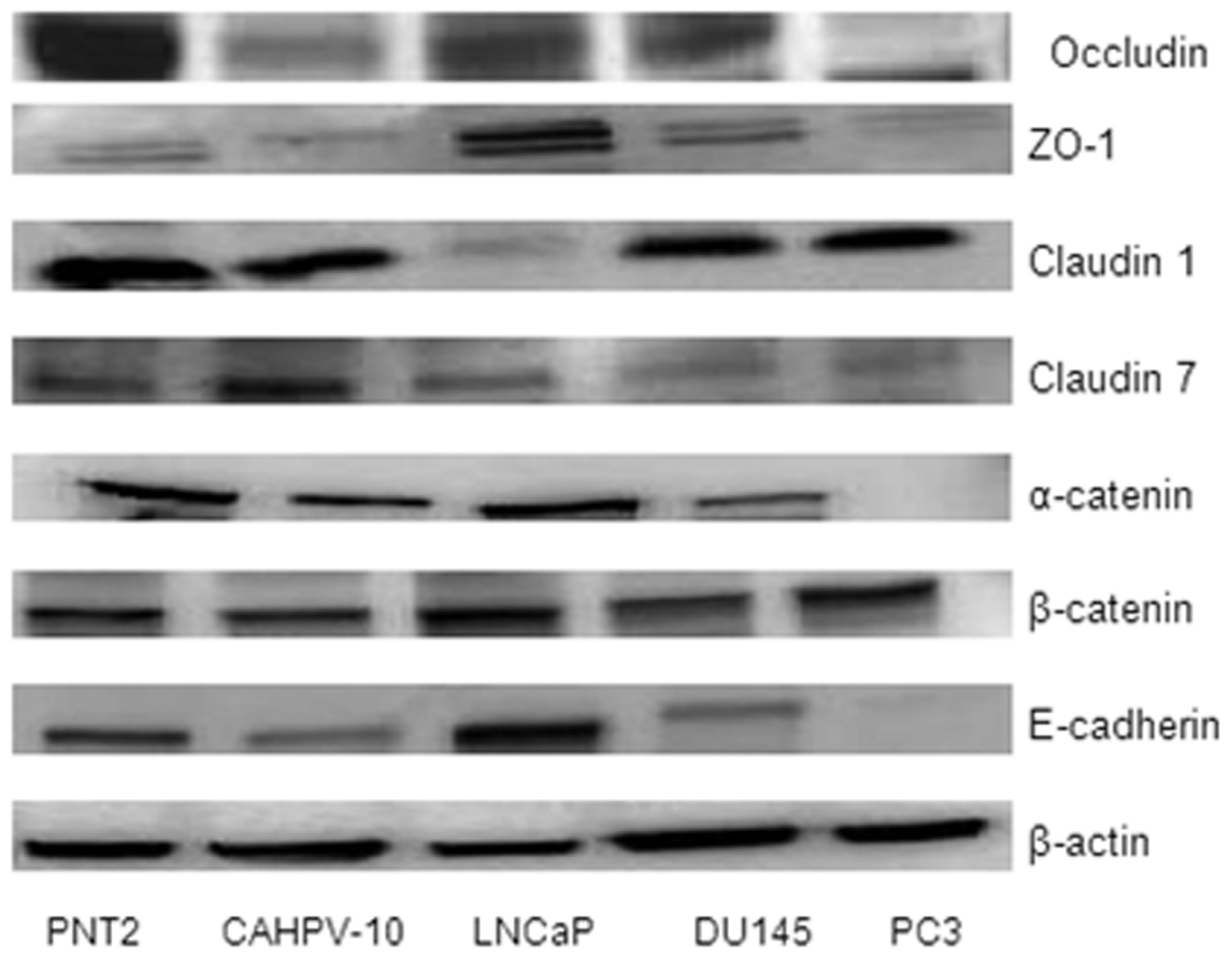
Western blot analysis demonstrating differential expression of Adherens and Tight junction protein expression in normal prostate cells (PNT2) and prostate cancer cell lines (CAHPV-10, LNCaP, DU145 and PC-3). β-actin was used as a loading control. Western blots were performed in duplicate and bands are representative of results obtained.

Western blot and densitometry were performed (Fig. 2) to ascertain whether the mRNA expression levels were comparable at the protein level (Table 2). Protein levels for occludin were down regulated in the prostate cancer cells CAHPV-10 (−2.8 fold), DU145 (−1.6 fold) and PC-3 (−9.3 fold) but up-regulated in LNCaP (2.4 fold) compared to PNT2. However, due to the high variation between replicates (data not shown), there was no significant difference between any of the prostate cells.

**Table 2:** mRNA expression and protein level fold changes compared to the normal prostate cells PNT2 for adherens (AJ) and tight junction (TJ) components.

	mRNA expression level	Protein level
**Occludin**		
CAHPV-10	*19.08	−2.8
LNCaP	*2.2	−2.4
DU145	*−3.2	−1.6
PC-3	*−4.3	−9.3
**ZO-1**		
CAHPV-10	1.31	1.1
LNCaP	1.28	3.6
DU145	1.26	2.5
PC-3	−1.20	−1.1
**Claudin 1**		
CAHPV-10	*−10	−2.1
LNCaP	*−100	*−9.3
DU145	**−2.1	−2.3
PC-3	**−3.8	−1.8
**Claudin 7**		
CAHPV-10	1.2	1.2
LNCaP	*4.63	*−1.1
DU145	**−5.3	*−1.1
PC-3	**−24.8	**−1.5
**α-catenin**		
CAHPV-10	−1.1	1
LNCaP	−1	**4.4
DU145	**−2.2	**−2
PC-3	**−33	n/a
**β-catenin**		
CAHPV-10	*−3	1.1
LNCaP	*−3.8	2
DU145	**2.2	1.4
PC-3	**2.1	1.5
**E-cadherin**		
CAHPV-10	*−2.3	**−1.7
LNCaP	*1.6	**2.7
DU145	*−5.6	**−1.5
PC-3	*−7.25	n/a

denotes significant difference (P<0.05) compared to PNT2 and

denotes a significant difference compared to the localised prostate cancer cells CAHPV-10. N/A denotes not applicable due to densitometry values not being calculated as bands were undetectable in those samples.

Protein levels for ZO-1 showed no significant different across any of the cell lines (CAHPV-10 1.1 fold, LNCaP 3.6-fold, DU145, 2.5 fold and PC-3 −1.1 fold).

Compared to PNT2, Claudin 1 levels were reduced in all prostate cancer cells. CAHPV-10 was down regulated −2.1 fold, DU145 −2.3 fold and PC-3 −1.8 fold. LNCaP was the only cell line to show a significant down-regulation compared to PNT2 (−9.3 fold, *p* = 0.04).

Claudin 7 protein levels showed no difference between CAHPV-10 (1.2 fold) and PNT2 but levels were significantly down regulated in LNCaP (−1.1 fold), DU145 (−1.1 fold) and PC-3 (−1.5 fold). All had a *p* = value of <0.01. However, LNCaP and DU145 did not meet the biological fold-change and therefore, these changes were not taken into account. In addition, when Claudin 7 protein levels for CAHPV-10 were compared to PC-3, a significant down-regulation was also documented (*p* = <0.01).

With regards to α- catenin protein levels, there was no significant difference between CAHPV-10 and PNT2, while for LNCaP (4.4 fold, *p* = 0.03), DU145 (−2 fold *p* = 0.04) α-catenin levels were significantly down regulated by various degrees when compared to PNT2. For, PC-3 a substantial down regulation in α - catenin protein levels was observed, such that a protein band was undetectable and a densitometry value could not be obtained. In addition, CAHPV-10 protein levels were also significantly different to LNCaP (*p* = 0.03) and DU145 (*p* = 0.04).

There was no significant difference in β-catenin protein levels between CAHPV-10 (1.1), LNCaP (2 fold), DU145 (1.4 fold) or PC-3 (1.5 fold) and PNT2, thus even though PC-3 exhibited the biological fold change, the result was disregarded.

For E-cadherin protein levels a significant down regulation was evident in CAHPV-10 (−1.7 fold, *p* = <0.01) and DU145 (−1.5 fold, *p* = <0.01). Again, a substantial down regulation in PC-3, such that a protein band was undetectable and a densitometry value could not be obtained. In LNCaP cells E-cadherin was up regulated at the protein level (2.7 fold *p* = 0.01).

### Immunofluorescence

In order to determine whether the AJ and TJ proteins were present at their correct sub-cellular location (cell membrane), immunofluorescence was utilised.

**Figure 3:**
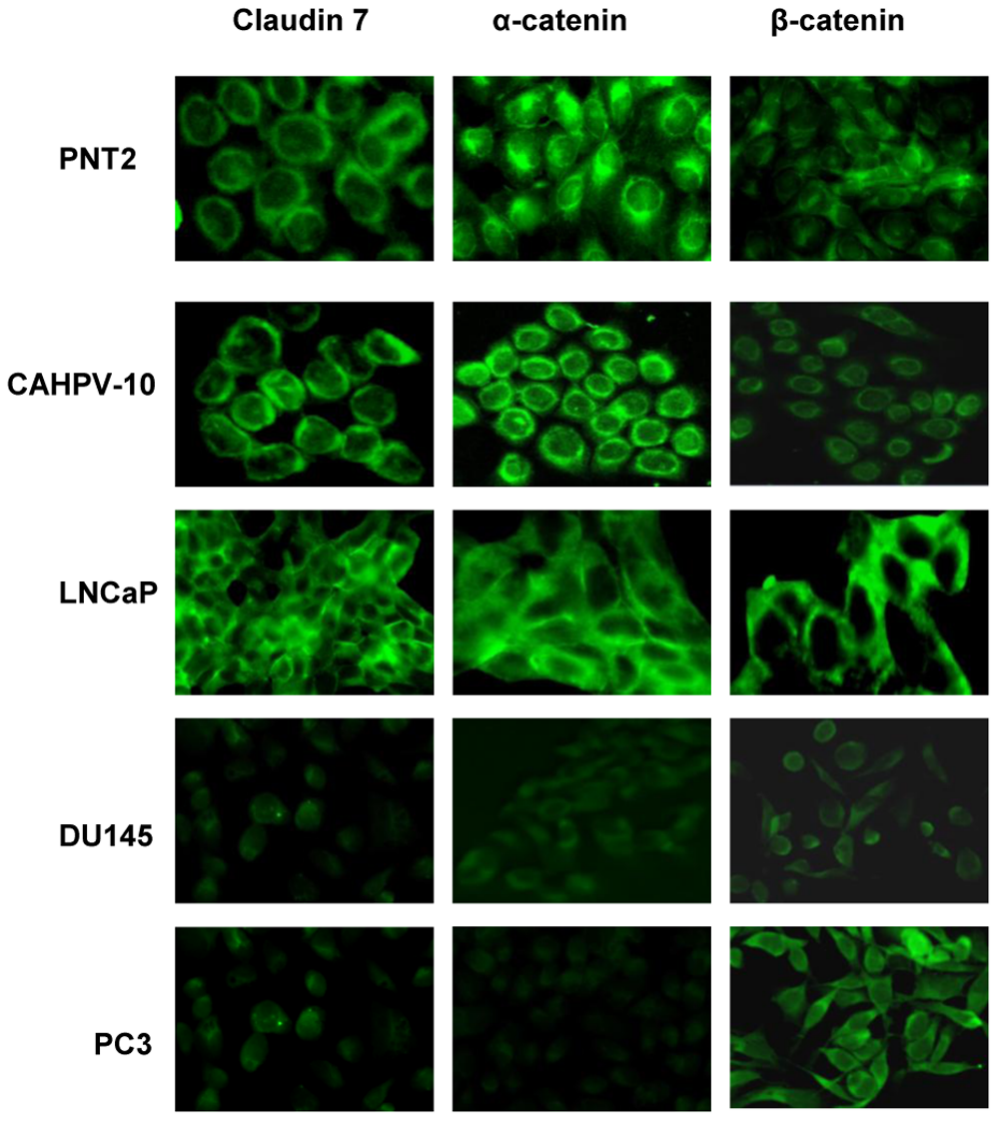
Fluorescence images of PNT2 and CAHPV-10 cells show membrane bound localisation of Claudin 7, α- catenin and β-catenin. For LNCaP cells, protein localisation occurs at the cell membrane and staining intensity appears similar to PNT2 (with the exception of β-catenin, which appears to have a higher expression). In DU145 cells, expression for all proteins is reduced and localisation is diffuse and cytoplasmic. For PC-3 cells, Claudin 7 and α- catenin, fluorescence is markedly reduced and/or cytoplasmic in distribution, while β-catenin fluorescence is stronger but also cytoplasmic in its localisation.

Immunofluorescence (Fig. 3) for Claudin 7 showed strong staining at the cell membrane for PNT2, CAHPV-10 and LNCaP. For both DU145 and PC-3, fluorescent staining was reduced. Staining was markedly reduced in both the DU145 and PC-3 cells with staining evident both at the cell membrane and in the cytoplasm. Staining for α- catenin showed strong specific staining at the cell membrane for PNT2, CAHPV-10 and LNCaP cells, while for DU145 and PC-3 signal intensity was markedly reduced and non specific. With regards to β-catenin fluorescence staining, PNT2, CAHPV-10 and DU145 exhibited specific staining at the cell membrane. β-catenin intensity in LNCaP and PC-3 cells was strong at the cell membrane and at sites of cell-cell contact, but in PC-3 staining was also evident in the cytoplasm.

## Discussion

The first step in the metastatic cascade is the loss of cell-cell adhesion in order for cells to break away from the primary tumour. Thus, AJs have been a major focus of cancer studies and now TJ components are also being recognised for their involvement. Alterations in key AJ and TJ components such as α-catenin, E-cadherin, β-catenin and Claudin 1 have been hailed as potential biomarkers for prostate cancer progression [18], [22], [23], [24] but the majority of research has been carried out on individual molecules. Cancer is heterogeneous by nature, thus there is a need for a panel of biomarkers, as opposed to single molecules, to help determine cancer progression and in the case of prostate cancer, distinguish dormant cancer from aggressive metastatic disease. As a result, the aim of this study was to evaluate the expression of several well known TJ and AJ components, collectively, as an important first step in identifying a panel of putative biomarkers that may help distinguish dormant prostate cancer from aggressive metastatic disease. Seven putative biomarkers were chosen due to their aberrant expression, which have been documented in the scientific literature and investigated in previous patient centred studies [17], [18], [20], [22], [23], [24], [25], [26], [27]. The 7 AJ and TJ proteins chosen were E-cadherin, α- and β- catenin, ZO-1, occludin, claudin 1 and claudin 7 and their expression was investigated using an *in vitro* model of prostate cancer progression. Out of the 7, we highlighted 3 (Claudin 7, α- and β- catenin) whose mRNA, protein levels and/or localization are similar in cells derived from both normal and organ-confined prostate cancer but their levels and/or localization all alter in cells derived from aggressive metastatic tumours. Claudin 7 mRNA and protein levels in non-invasive prostate cancer cells (CAHPV-10) were similar to those found in prostate epithelial cells. In addition both mRNA and protein levels were significantly down regulated in the most aggressive metastatic cell line PC-3 when compared to CAHPV-10. These results are supported by Sheehan et al, who showed a decrease in Claudin 7 expression correlated with high grade prostatic tumours [19]. Therefore, Claudin 7 protein levels may be a reflection of the aggressiveness of these prostate cancer cells and may only be altered in the most aggressive form of the disease. In addition, further analysis by immunofluorescence revealed that Claudin 7 localisation in the PC-3 cells was cytoplasmic as well as membrane bound. Aberrant localisation of Claudin 7 to the cytoplasm has been shown to occur in breast cancer cells [28] and oesophageal squamous cell carcinoma [2]. This re-distribution of Claudins have been attributed to several mechanism including protein down regulation resulting in cytoplasmic internalisation [2]. Based on these results it may be hypothesised that Claudin 7 could be a possible candidate for distinguishing indolent cancers from the most aggressive metastatic forms, which requires validation in patient samples.

In combination with Claudin 7, α -catenin levels in non-invasive prostate cancer cells were similar to levels in prostate epithelial cells, but like Claudin 7, was significantly decreased in the most aggressive metastatic cells. A significant relationship between reduced α-catenin expression and high gleason score has been reported in prostate tumours [24]. Similarly, α-catenin has been reported to be present in prostate tumours with a low gleason grade [17]. It has been suggested, therefore, that loss of α-catenin leads to the loss of e-cadherin function leading to a poorer prognosis [18], whereas, repletion of α-catenin in α-catenin null prostate cancer cells has been reported to increase adherens junction formation and reduced transcriptional activity of β-catenin, cyclin D1 levels and cell proliferation [26].

For β-catenin mRNA protein levels and immunofluorescent staining intensity of CAHPV-10 cells were similar to normal prostate epithelial cells and interestingly, immunofluorescence revealed β-catenin in PC-3 cells to be both membrane and cytoplasmic. β-catenin localises to the cytoplasm as a result of adherens junction complex breakdown and then translocates to the nucleus where it exerts it transcriptional effect [29]. This aberrant localisation to the cytoplasm has been shown to contribute to thyroid carciongenesis [30] and linked to a poor prognosis in breast cancer patients [31]. Our results suggest that it may be pertinent to look at cytoplasmic localisation of β-catenin, as opposed to quantitatively assessing it's mRNA or protein levels, as a putative biomarker of aggressive disease.

It should be noted that the mRNA and protein expression levels in the LNCaP cell line rarely followed the same trend as DU145 and PC-3. This difference in expression levels could be explained by the metastatic potential and differentiation status of the cell line. LNCaP has been documented as having limited metastatic potential and remains well differentiated when introduced into mouse models [32]. Thus it may be postulated that following metastasis to the lymph nodes, this cell line has reverted back to a more epithelial form as a result of colonisation [33] and growth. In addition, there were also some discrepancies between mRNA and protein levels for Claudin 7 and occludin in LNCaP, and β-catenin in CAHPV −10 and DU145 and α-catenin in CAHPV-10 and LNCaP. As mRNA is translated into protein it can be assumed that there should be a correlation between mRNA and protein levels. However, studies that have investigated a correlation between mRNA and protein expression levels have reported minimal or limited correlations [34]. The amount of protein detected may likely be affected by translation and post-transcriptional modifications providing a secondary degree of expression control that could account for these differences.

## Conclusion

Due to the heterogeneous nature of cancer, analysis of single molecules provides limited information and it is impossible to diagnose cancer or predict disease progression using a single biomarker. The 7 biomarkers analysed here, have been reported to be aberrantly expressed in prostate cancer tissue samples but mainly on an individual basis. However, our *in vitro* investigation has shown, that out of these 7 only 3 (Claudin 7, α-catenin and β-catenin) may collectively be used to distinguish localised prostate cancer from cells representing aggressive metastatic disease. We believe that this *in vitro* identification of alterations in several key genes in combination highlights the importance of utilising several molecular markers and is an important first step in identifying a panel of putative biomarkers that may help distinguish dormant prostate cancer from aggressive metastatic disease. A large patient IHC study is now needed to validate these results.
